# Supplementary material for: Hemodynamic molecular imaging of tumor-associated enzyme activity in the living brain
Source: eLife. 2021 Dec 21;10:e70237. doi: 10.7554/eLife.70237 (PMC8691830; doi:10.7554/eLife.70237)
Supplement: Supplementary file 1. [file elife-70237-supp1.docx]

**Supplementary File 1.** Sequences of candidate FAP probes

| # | Peptide sequence* |
| --- | --- |
| **1** | Biotin-TSGPNQA**C**DTAT**C**VTHRLAGLLSRSGGVVKNNFVPTNVGSKAF |
| **2** | Biotin-TSGPNQEQEA**C**DTAT**C**VTHRLAGLLSRSGGVVKNNFVPTNVGSKAF |
| **3** | Biotin-SSGPVAA**C**DTAT**C**VTHRLAGLLSRSGGVVKNNFVPTNVGSKAF |
| **4** | Biotin-SSGPVADGA**C**DTAT**C**VTHRLAGLLSRSGGVVKNNFVPTNVGSKAF |
| **5** | Biotin-ASGPAGPAA**C**DTAT**C**VTHRLAGLLSRSGGVVKNNFVPTNVGSKAF |

* Color coding: cyan = CGRP sequence; red = FAP dipeptide recognition sites.
