# Supplementary material for: Hemodynamic molecular imaging of tumor-associated enzyme activity in the living brain
Source: eLife. 2021 Dec 21;10:e70237. doi: 10.7554/eLife.70237 (PMC8691830; doi:10.7554/eLife.70237)
Supplement: Supplementary file 2. [file elife-70237-supp2.docx]

**Supplementary File 2.** FAP expression plasmid sequence

Color coding: gray = hEF1α promoter; red = human FAP gene; purple = IRES; magenta = mKate gene; green = blasticidin resistance gene.

1 tgcctttgca gctaatggac cttctaggtc ttgaaaggag tgggaattgg ctccggtgcc

61 cgtcagtggg cagagcgcac atcgcccaca gtccccgaga agttgggggg aggggtcggc

121 aattgaaccg gtgcctagag aaggtggcgc ggggtaaact gggaaagtga tgtcgtgtac

181 tggctccgcc tttttcccga gggtggggga gaaccgtata taagtgcagt agtcgccgtg

241 aacgttcttt ttcgcaacgg gtttgccgcc agaacacagg taagtgccgt gtgtggttcc

301 cgcgggcctg gcctctttac gggttatggc ccttgcgtgc cttgaattac ttccacctgg

361 ctgcagtacg tgattcttga tcccgagctt cgggttggaa gtgggtggga gagttcgagg

421 ccttgcgctt aaggagcccc ttcgcctcgt gcttgagttg aggcctggcc tgggcgctgg

481 ggccgccgcg tgcgaatctg gtggcacctt cgcgcctgtc tcgctgcttt cgataagtct

541 ctagccattt aaaatttttg atgacctgct gcgacgcttt ttttctggca agatagtctt

601 gtaaatgcgg gccaagatct gcacactggt atttcggttt ttggggccgc gggcggcgac

661 ggggcccgtg cgtcccagcg cacatgttcg gcgaggcggg gcctgcgagc gcggccaccg

721 agaatcggac gggggtagtc tcaagctggc cggcctgctc tggtgcctgg cctcgcgccg

781 ccgtgtatcg ccccgccctg ggcggcaagg ctggcccggt cggcaccagt tgcgtgagcg

841 gaaagatggc cgcttcccgg ccctgctgca gggagctcaa aatggaggac gcggcgctcg

901 ggagagcggg cgggtgagtc acccacacaa aggaaaaggg cctttccgtc ctcagccgtc

961 gcttcatgtg actccacgga gtaccgggcg ccgtccaggc acctcgatta gttctcgagc

1021 ttttggagta cgtcgtcttt aggttggggg gaggggtttt atgcgatgga gtttccccac

1081 actgagtggg tggagactga agttaggcca gcttggcact tgatgtaatt ctccttggaa

1141 tttgcccttt ttgagtttgg atcttggttc attctcaagc ctcagacagt ggttcaaagt

1201 ttttttcttc catttcaggt gtcgtgagga attagcttgg tactaatacg actcactata

1261 gcctggccac catgaagact tgggtaaaaa tcgtatttgg agttgccacc tctgctgtgc

1321 ttgccttatt ggtgatgtgc attgtcttac gcccttcaag agttcataac tctgaagaaa

1381 atacaatgag agcactcaca ctgaaggata ttttaaatgg aacattttct tataaaacat

1441 tttttccaaa ctggatttca ggacaagaat atcttcatca atctgcagat aacaatatag

1501 tactttataa tattgaaaca ggacaatcat ataccatttt gagtaataga accatgaaaa

1561 gtgtgaatgc ttcaaattac ggcttatcac ctgatcggca atttgtatat ctagaaagtg

1621 attattcaaa gctttggaga tactcttaca cagcaacata ttacatctat gaccttagca

1681 atggagaatt tgtaagagga aatgagcttc ctcgtccaat tcagtattta tgctggtcgc

1741 ctgttgggag taaattagca tatgtctatc aaaacaatat ctatttgaaa caaagaccag

1801 gagatccacc ttttcaaata acatttaatg gaagagaaaa taaaatattt aatggaatcc

1861 cagactgggt ttatgaagag gaaatgcttg ctacaaaata tgctctctgg tggtcgccta

1921 atggaaaatt tttggcatat gcggaattta atgatacgga tataccagtt attgcctatt

1981 cctattatgg cgatgaacaa tatcctagaa caataaatat tccataccca aaggctggag

2041 ctaagaatcc cgttgttcgg atatttatta tcgataccac ttaccctgcg tatgtaggtc

2101 cccaggaagt gcctgttcca gcaatgatag cctcaagtga ttattatttc agttggctca

2161 cgtgggttac tgatgaacga gtatgtttgc agtggctaaa aagagtccag aatgtttcgg

2221 tcctgtctat atgtgacttc agggaagact ggcagacatg ggattgtcca aagacccagg

2281 agcatataga agaaagcaga actggatggg ctggtggatt ctttgtttca acaccagttt

2341 tcagctatga tgccatttcg tactacaaaa tatttagtga caaggatggc tacaaacata

2401 ttcactatat caaagacact gtggaaaatg ctattcaaat tacaagtggc aagtgggagg

2461 ccataaatat attcagagta acacaggatt cactgtttta ttctagcaat gaatttgaag

2521 aataccctgg aagaagaaac atctacagaa ttagcattgg aagctatcct ccaagcaaga

2581 agtgtgttac ttgccatcta aggaaagaaa ggtgccaata ttacacagca agtttcagcg

2641 actacgccaa gtactatgca cttgtctgct acggcccagg catccccatt tccacccttc

2701 atgatggacg cactgatcaa gaaattaaaa tcctggaaga aaacaaggaa ttggaaaatg

2761 ctttgaaaaa tatccagctg cctaaagagg aaattaagaa acttgaagta gatgaaatta

2821 ctttatggta caagatgatt cttcctcctc aatttgacag atcaaagaag tatcccttgc

2881 taattcaagt gtatggtggt ccctgcagtc agagtgtaag gtctgtattt gctgttaatt

2941 ggatatctta tcttgcaagt aaggaaggga tggtcattgc cttggtggat ggtcgaggaa

3001 cagctttcca aggtgacaaa ctcctctatg cagtgtatcg aaagctgggt gtttatgaag

3061 ttgaagacca gattacagct gtcagaaaat tcatagaaat gggtttcatt gatgaaaaaa

3121 gaatagccat atggggctgg tcctatggag gatacgtttc atcactggcc cttgcatctg

3181 gaactggtct tttcaaatgt ggtatagcag tggctccagt ctccagctgg gaatattacg

3241 cgtctgtcta cacagagaga ttcatgggac tcccaacaaa ggatgataat cttgagcact

3301 ataagaattc aactgtgatg gcaagagcag aatatttcag aaatgtagac tatcttctca

3361 tccacggaac agcagatgat aatgtgcact ttcaaaactc agcacagatt gctaaagctc

3421 tggttaatgc acaagtggat ttccaggcaa tgtggtactc tgaccagaac cacggcttat

3481 ccggcctgtc cacgaaccac ttatacaccc acatgaccca cttcctaaag cagtgtttct

3541 ctttgtcaga ctaagactgg gatccgcccc tctccctccc ccccccctaa cgttactggc

3601 cgaagccgct tggaataagg ccggtgtgcg tttgtctata tgttattttc caccatattg

3661 ccgtcttttg gcaatgtgag ggcccggaaa cctggccctg tcttcttgac gagcattcct

3721 aggggtcttt cccctctcgc caaaggaatg caaggtctgt tgaatgtcgt gaaggaagca

3781 gttcctctgg aagcttcttg aagacaaaca acgtctgtag cgaccctttg caggcagcgg

3841 aaccccccac ctggcgacag gtgcctctgc ggccaaaagc cacgtgtata agatacacct

3901 gcaaaggcgg cacaacccca gtgccacgtt gtgagttgga tagttgtgga aagagtcaaa

3961 tggctctcct caagcgtatt caacaagggg ctgaaggatg cccagaaggt accccattgt

4021 atgggatctg atctggggcc tcggtacaca tgctttacat gtgtttagtc gaggttaaaa

4081 aaacgtctag gccccccgaa ccacggggac gtggttttcc tttgaaaaac acgatgataa

4141 tatggccaca gccaccatgg tgagcgagct gattaaggag aacatgcaca tgaagctgta

4201 catggagggc accgtgaaca accaccactt caagtgcaca tccgagggcg aaggcaagcc

4261 ctacgagggc acccagacca tgagaatcaa ggcggtcgag ggcggccctc tccccttcgc

4321 cttcgacatc ctggctacca gcttcatgta cggcagcaaa accttcatca accacaccca

4381 gggcatcccc gacttcttta agcagtcctt ccccgagggc ttcacatggg agagagtcac

4441 cacatacgaa gatgggggcg tgctgaccgc tacccaggac accagcctcc aggacggctg

4501 cctcatctac aacgtcaaga tcagaggggt gaacttccca tccaacggcc ctgtgatgca

4561 gaagaaaaca ctcggctggg aggcctccac cgagacactg taccccgctg acggcggcct

4621 ggaaggcaga gccgacatgg ccctgaagct cgtgggcggg ggccacctga tctgcaacct

4681 taagaccaca tacagatcca agaaacccgc taagaacctc aagatgcccg gcgtctacta

4741 tgtggacagg agactggaaa gaatcaagga ggccgacaaa gagacatacg tcgagcagca

4801 cgaggtggct gtggccagat actgcgacct ccctagcaaa ctggggcaca aacttaattc

4861 cgctgagggc cgcggcagcc tgctgacctg cggcgacgtg gaggaaaacc caggcccaat

4921 ggctaagcct ttgtctcaag aagaatccac cctcattgaa agagcaacgg ctacaatcaa

4981 cagcatcccc atctctgaag actacagcgt cgccagcgca gctctctcta gcgacggccg

5041 catcttcact ggtgtcaatg tatatcattt tactggggga ccttgtgcag aactcgtggt

5101 gctgggcact gctgctgctg cggcagctgg caacctgact tgtatcgtcg cgatcggaaa

5161 tgagaacagg ggcatcttga gcccctgcgg acggtgccga caggtgcttc tcgatctgca

5221 tcctgggatc aaagccatag tgaaggacag tgatggacag ccgacggcag ttgggattcg

5281 tgaattgctg ccctctggtt atgtgtggga gggataaggg acaggtgata tccagcacag

5341 tggcggccgc tcgacaatca acctctggat tacaaaattt gtgaaagatt gactggtatt

5401 cttaactatg ttgctccttt tacgctatgt ggatacgctg ctttaatgcc tttgtatcat

5461 gctattgctt cccgtatggc tttcattttc tcctccttgt ataaatcctg gttgctgtct

5521 ctttatgagg agttgtggcc cgttgtcagg caacgtggcg tggtgtgcac tgtgtttgct

5581 gacgcaaccc ccactggttg gggcattgcc accacctgtc agctcctttc cgggactttc

5641 gctttccccc tccctattgc cacggcggaa ctcatcgccg cctgccttgc ccgctgctgg

5701 acaggggctc ggctgttggg cactgacaat tccgtggtgt tgtcggggaa gctgacgtcc

5761 tttccatggc tgctcgcctg tgttgccacc tggattctgc gcgggacgtc cttctgctac

5821 gtcccttcgg ccctcaatcc agcggacctt ccttcccgcg gcctgctgcc ggctctgcgg

5881 cctcttccgc gtcttcgcct tcgccctcag acgagtcgga tctccctttg ggccgcctcc

5941 ccgcctggaa ttctgcagat atccggttag taatgagttt ggaattaatt ctgtggaatg

6001 tgtgtcagtt agggtgtgga aagtccccag gctccccagg caggcagaag tatgcaaagc

6061 atgcatctca attagtcagc aaccaggtgt ggaaagtccc caggctcccc agcaggcaga

6121 agtatgcaaa gcatgcatct caattagtca gcaaccatag tcccgcccct aactccgccc

6181 atcccgcccc taactccgcc cagttccgcc cattctccgc cccatggctg actaattttt

6241 tttatttatg cagaggccga ggccgcctct gcctctgagc tattccagaa gtagtgagga

6301 ggcttttttg gaggcctagg cttttgcaaa aagctccccc tgttgacaat taatcatcgg

6361 catagtatat cggcatagta taatacgaca aggtgaggaa ctaaaccatg gccaagttga

6421 ccagtgccgt tccggtgctc accgcgcgcg acgtcgccgg agcggtcgag ttctggaccg

6481 accggctcgg gttctcccgg gacttcgtgg aggacgactt cgccggtgtg gtccgggacg

6541 acgtgaccct gttcatcagc gcggtccagg accaggtggt gccggacaac accctggcct

6601 gggtgtgggt gcgcggcctg gacgagctgt acgccgagtg gtcggaggtc gtgtccacga

6661 acttccggga cgcctccggg ccggccatga ccgagatcgg cgagcagccg tgggggcggg

6721 agttcgccct gcgcgacccg gccggcaact gcgtgcactt cgtggccgag gagcaggact

6781 gacacgtgct acgagattta aatggtacct ttaagaccaa tgacttacaa ggcagctgta

6841 gatcttagcc actttttaaa agaaaagggg ggactggaag ggctagctca ctcccaacga

6901 agacaagatc tgctttttgc ttgtactggg tctctctggt tagaccagat ctgagcctgg

6961 gagctctctg gctgcctagg gaacccactg cttaagcctc aataaagctt gccttgagtg

7021 cttcaagtag tgtgtgcccg tctgttgtgt gactctggta actagagatc cctcagaccc

7081 ttttagtcag tgtggaaaat ctctagcagt agtagttcat gtcatcttat tattcagtat

7141 ttataacttg caaagaaatg aatatcagag agtgagagga acttgtttat tgcagcttat

7201 aatggttaca aataaagcaa tagcatcaca aatttcacaa ataaagcatt tttttcactg

7261 cattctagtt gtggtttgtc caaactcatc aatgtatctt atcatgtctg gctctagcta

7321 tcccgcccct aactccgccc agttccgccc attctccgcc ccatggctga ctaatttttt

7381 ttatttatgc agaggccgag gccgacgtgt gtttcttaga cgtcaggtgg cacttttcgg

7441 ggaaatgtgc gcggaacccc tatttgttta tttttctaaa tacattcaaa tatgtatccg

7501 ctcatgagac aataaccctg ataaatgctt caataatatt gaaaaaggaa gagtatgagt

7561 attcaacatt tccgtgtcgc ccttattccc ttttttgcgg cattttgcct tcctgttttt

7621 gctcacccag aaacgctggt gaaagtaaaa gatgctgaag atcagttggg tgcacgagtg

7681 ggttacatcg aactggatct caacagcggt aagatccttg agagttttcg ccccgaagaa

7741 cgttttccaa tgatgagcac ttttaaagtt ctgctatgtg gcgcggtatt atcccgtatt

7801 gacgccgggc aagagcaact cggtcgccgc atacactatt ctcagaatga cttggttgag

7861 tactcaccag tcacagaaaa gcatcttacg gatggcatga cagtaagaga attatgcagt

7921 gctgccataa ccatgagtga taacactgcg gccaacttac ttctgacaac gatcggagga

7981 ccgaaggagc taaccgcttt tttgcacaac atgggggatc atgtaactcg ccttgatcgt

8041 tgggaaccgg agctgaatga agccatacca aacgacgagc gtgacaccac gatgcctgta

8101 gcaatggcaa caacgttgcg caaactatta actggcgaac tacttactct agcttcccgg

8161 caacaattaa tagactggat ggaggcggat aaagttgcag gaccacttct gcgctcggcc

8221 cttccggctg gctggtttat tgctgataaa tctggagccg gtgagcgtgg gtctcgcggt

8281 atcattgcag cactggggcc agatggtaag ccctcccgta tcgtagttat ctacacgacg

8341 gggagtcagg caactatgga tgaacgaaat agacagatcg ctgagatagg tgcctcactg

8401 attaagcatt ggtaactgtc agaccaagtt tactcatata tactttagat tgatttaaaa

8461 cttcattttt aatttaaaag gatctaggtg aagatccttt ttgataatct catgaccaaa

8521 atcccttaac gtgagttttc gttccactga gcgtcagacc ccgtagaaaa gatcaaagga

8581 tcttcttgag atcctttttt tctgcgcgta atctgctgct tgcaaacaaa aaaaccaccg

8641 ctaccagcgg tggtttgttt gccggatcaa gagctaccaa ctctttttcc gaaggtaact

8701 ggcttcagca gagcgcagat accaaatact gtccttctag tgtagccgta gttaggccac

8761 cacttcaaga actctgtagc accgcctaca tacctcgctc tgctaatcct gttaccagtg

8821 gctgctgcca gtggcgataa gtcgtgtctt accgggttgg actcaagacg atagttaccg

8881 gataaggcgc agcggtcggg ctgaacgggg ggttcgtgca cacagcccag cttggagcga

8941 acgacctaca ccgaactgag atacctacag cgtgagctat gagaaagcgc cacgcttccc

9001 gaagggagaa aggcggacag gtatccggta agcggcaggg tcggaacagg agagcgcacg

9061 agggagcttc cagggggaaa cgcctggtat ctttatagtc ctgtcgggtt tcgccacctc

9121 tgacttgagc gtcgattttt gtgatgctcg tcaggggggc ggagcctatg gaaaaacgcc

9181 agcaacgcgg cctttttacg gttcctggcc ttttgctggc cttttgctca catgttcttt

9241 cctgcgttat cccctgattc tgtggataac cgtattaccg cctttgagtg agctgatacc

9301 gctcgccgca gccgaacgac cgagcgcagc gagtcagtga gcgaggaagc ggaagagcgc

9361 ccaatacgca aaccgcctct ccccgcgcgt tggccgattc attaatgcag ctggcacgac

9421 aggtttcccg actggaaagc gggcagtgag cgcaacgcaa ttaatgtgag ttagctcact

9481 cattaggcac cccaggcttt acactttatg cttccggctc gtatgttgtg tggaattgtg

9541 agcggataac aatttcacac aggaaacagc tatgaccatg attacgccaa gcgcgcaatt

9601 aaccctcact aaagggaaca aaagctggag ctgcaagctt aatgtagtct tatgcaatac

9661 tcttgtagtc ttgcaacatg gtaacgatga gttagcaaca tgccttacaa ggagagaaaa

9721 agcaccgtgc atgccgattg gtggaagtaa ggtggtacga tcgtgcctta ttaggaaggc

9781 aacagacggg tctgacatgg attggacgaa ccactgaatt gccgcattgc agagatattg

9841 tatttaagtg cctagctcga tacaataaac gggtctctct ggttagacca gatctgagcc

9901 tgggagctct ctggctaact agggaaccca ctgcttaagc ctcaataaag cttgccttga

9961 gtgcttcaag tagtgtgtgc ccgtctgttg tgtgactctg gtaactagag atccctcaga

10021 cccttttagt cagtgtggaa aatctctagc agtggcgccc gaacagggac ctgaaagcga

10081 aagggaaacc agagctctct cgacgcagga ctcggcttgc tgaagcgcgc acggcaagag

10141 gcgaggggcg gcgactggtg agtacgccaa aaattttgac tagcggaggc tagaaggaga

10201 gagatgggtg cgagagcgtc agtattaagc gggggagaat tagatcgcga tgggaaaaaa

10261 ttcggttaag gccaggggga aagaaaaaat ataaattaaa acatatagta tgggcaagca

10321 gggagctaga acgattcgca gttaatcctg gcctgttaga aacatcagaa ggctgtagac

10381 aaatactggg acagctacaa ccatcccttc agacaggatc agaagaactt agatcattat

10441 ataatacagt agcaaccctc tattgtgtgc atcaaaggat agagataaaa gacaccaagg

10501 aagctttaga caagatagag gaagagcaaa acaaaagtaa gaccaccgca cagcaagcgg

10561 ccgctgatct tcagacctgg aggaggagat atgagggaca attggagaag tgaattatat

10621 aaatataaag tagtaaaaat tgaaccatta ggagtagcac ccaccaaggc aaagagaaga

10681 gtggtgcaga gagaaaaaag agcagtggga ataggagctt tgttccttgg gttcttggga

10741 gcagcaggaa gcactatggg cgcagcctca atgacgctga cggtacaggc cagacaatta

10801 ttgtctggta tagtgcagca gcagaacaat ttgctgaggg ctattgaggc gcaacagcat

10861 ctgttgcaac tcacagtctg gggcatcaag cagctccagg caagaatcct ggctgtggaa

10921 agatacctaa aggatcaaca gctcctgggg atttggggtt gctctggaaa actcatttgc

10981 accactgctg tgccttggaa tgctagttgg agtaataaat ctctggaaca gattggaatc

11041 acacgacctg gatggagtgg gacagagaaa ttaacaatta cacaagctta atacactcct

11101 taattgaaga atcgcaaaac cagcaagaaa agaatgaaca agaattattg gaattagata

11161 aatgggcaag tttgtggaat tggtttaaca taacaaattg gctgtggtat ataaaattat

11221 tcataatgat agtaggaggc ttggtaggtt taagaatagt ttttgctgta ctttctatag

11281 tgaatagagt taggcaggga tattcaccat tatcgtttca gacccacctc ccaaccccga

11341 ggggacccga caggcccgaa ggaatagaag aagaaggtgg agagagagac agagacagat

11401 ccattcgatt agtgaacgga tctcgacggt taacttttaa aagaaaaggg gggattgggg

11461 ggtacagtgc aggggaaaga atagtagaca taatagcaac agacatacaa actaaagaat

11521 tacaaaaaca aattacaaaa attcaaaatt ttattccagt gtggtggaat tctgcagtc
